# Development and Application of a Fast Method to Acquire the Accurate Whole-Genome Sequences of Human Adenoviruses

**DOI:** 10.3389/fmicb.2021.661382

**Published:** 2021-05-14

**Authors:** Shan Zhao, Wenyi Guan, Kui Ma, Yuqian Yan, Junxian Ou, Jing Zhang, Zhiwu Yu, Jianguo Wu, Qiwei Zhang

**Affiliations:** ^1^Guangdong Provincial Key Laboratory of Tropical Disease Research, School of Public Health, Southern Medical University, Guangzhou, China; ^2^Guangdong Provincial Key Laboratory of Virology, Institute of Medical Microbiology, Jinan University, Guangzhou, China; ^3^Division of Laboratory Science, Affiliated Cancer Hospital & Institute of Guangzhou Medical University, Guangzhou, China

**Keywords:** whole-genome sequencing, genomic DNA, human adenovirus, human adenovirus type 14, human adenovirus type 2, human adenovirus type 4, Sanger sequencing, inverted terminal repeats (ITRs)

## Abstract

The whole-genome sequencing (WGS) of human adenoviruses (HAdVs) plays an important role in identifying, typing, and mutation analysis of HAdVs. Nowadays, three generations of sequencing have been developed. The accuracy of first-generation sequencing is up to 99.99%, whereas this technology relies on PCR and is time consuming; the next-generation sequencing (NGS) is expensive and not cost effective for determining a few special samples; and the third-generation sequencing technology has a higher error rate. In this study, first, we developed an efficient HAdV genomic DNA extraction method. Using the complete genomic DNA instead of the PCR amplicons as the direct sequencing template and a set of walking primers, we developed the HAdV WGS method based on first-generation sequencing. The HAdV whole genomes were effectively sequenced by a set of one-way sequencing primers designed, which reduced the sequencing time and cost. More importantly, high sequence accuracy is guaranteed. Four HAdV strains (GZ01, GZ02, HK35, and HK91) were isolated from children with acute respiratory diseases (ARDs), and the complete genomes were sequenced using this method. The accurate sequences of the whole inverted terminal repeats (ITRs) at both ends of the HAdV genomes were also acquired. The genome sequence of human adenovirus type 14 (HAdV-B14) strain GZ01 acquired by this method is identical to the sequence released in GenBank, which indicates that this novel sequencing method has high accuracy. The comparative genomic analysis identified that strain GZ02 isolated in September 2010 had the identical genomic sequence with the HAdV-B14 strain GZ01 (October 2010). Therefore, strain GZ02 is the first HAdV-B14 isolate emergent in China (September 2010; GenBank acc no. MW692349). The WGS of HAdV-C2 strain HK91 and HAdV-E4 strain HK35 isolated from children with acute respiratory disease in Hong Kong were also determined by this sequencing method. In conclusion, this WGS method is fast, accurate, and universal for common human adenovirus species B, C, and E. The sequencing strategy may also be applied to the WGS of the other DNA viruses.

## Introduction

Human adenoviruses (HAdVs) have seven species, A (HAdV-A) through G (HAdV-G), defined by various biological and morphological criteria, nucleic acid characteristics, and homologies (Zhang et al., 2017; Cheng et al., 2018). One-third of HAdVs are related to human diseases and are estimated to cause 8% of global clinically relevant viral diseases (Yu et al., 2016). Common adenoviral diseases include respiratory infections in children and military recruits, infantile gastroenteritis, and ocular infections among healthy individuals (Zhang et al., 2009, 2012b; Han et al., 2013). Less frequently, these pathogens can cause urinary tract infections, myocarditis, meningoencephalitis, and acute hemorrhagic cystitis. Meanwhile, in neonates and immunocompromised individuals, HadVs have been reported to cause fulminant fatal pneumonia, hepatitis, and/or encephalitis (Adhikary et al., 2014; Scott et al., 2016; Cheng et al., 2018). In 2019, a local outbreak of adenovirus in southwest China killed more than 40 children.

Human adenoviruses are usually classified by serological criteria and hemagglutination-inhibition tests, both of which are associated with the three major capsid proteins, hexon, fiber, and penton base (Zhang et al., 2009; Seto et al., 2010). But now, the classification of adenovirus is usually based on more modern criteria, including genome and phylogenetic analysis (Robinson et al., 2013), which uses the whole-genome sequence to characterize and name human adenoviruses (Human Adenovirus Working Group (HAWG)^1^ (Seto et al., 2011). The commonly used sequencing methods include the Sanger sequencing method and the next- and third-generation sequencing technology. The traditional first-generation sequencing technology represented by Sanger sequencing reads up as long as 1,000 bp, and the accuracy is as high as 99.99% (Sanger et al., 1977; Lu et al., 2016; Shendure et al., 2017). However, the first-generation sequencing technology takes much time because it depends on polymerase chain reactions (PCRs) and/or DNA electrophoresis separation technology. The next-generation sequencing (NGS) technology is expensive and not suitable for the common laboratories in developing countries to determine large clinical samples’ whole-genome sequences (Koboldt et al., 2013; Lu et al., 2016; Shendure et al., 2017). Currently, more than 95% of next-generation sequencing (NGS) applications require the reads aligning to a reference genome or reference transcriptome sequence, termed “mapping.” However, the accuracy of mapping varies considerably, and mutations or recombinations are often missed or ignored (Zhang et al., 2021); the third-generation sequencing technology has a high error rate (Lu et al., 2016; Shendure et al., 2017; van Dijk et al., 2018). Recently, a new sequencing technology, nanopore sequencing, had been developed, which was famous for low cost and little sample preparation, but irregular steps caused substantial errors by the motor enzymes (Rusk, 2019).

The next-generation whole-genome sequencing is classified into *de novo* sequencing and resequencing. *De novo* sequencing does not require any reference genome information or custom primers. Bioinformatics analysis methods are used to splice and assemble the genome. However, *de novo* sequencing is easy to produce gaps, requiring primer design and PCR to obtain the gap sequences. Additionally, there are more mismatches than one generation. Resequencing is the sequencing of the genomes with reference genomic sequences from the same or similar species. However, this strategy works badly for recombinant viruses. For example, internal recombination within human adenovirus species B is so common that the reference strain is not easy to select correctly (Zhang et al., 2016). As a result, the recombination or insertions/deletions (indels) might be mistaken for mismatches and ignored, failing to identify the recombination or indels in clinical isolates.

The lack of accuracy is still a major weakness of NGS when compared with first-generation sequencing. This study improved the sequencing method based on the first-generation sequencing and applied it in the clinical sample sequencing. First, we developed an effective adenovirus genomic DNA extraction method. Using the adenovirus genomic DNA as a direct sequencing template instead of the PCR amplicons, we did the Sanger sequencing using a set of walking primers covering the whole genome. This method reduces the mismatches and time without any PCR procedure and also brings down the costs. Second, we obtained the whole-genomic sequences of four HAdV isolates [strains GZ01 (HAdV-B14), GZ02 (HAdV-B14), HK91 (HAdV-C2), and HK35 (HAdV-E4)], with both complete inverted terminal repeat (ITR) ends sequenced successfully using this method. The genome sequence of strain GZ01 acquired by this method is identical to the sequence released before (GenBank no. JQ824845), which indicates that this novel sequencing method has high accuracy. We also found that strain GZ02 isolated in September 2010 had the identical genomic sequence with strain GZ01 isolated in October 2010 (Zhang et al., 2012b, 2017), which suggests that both isolates derived from the same strain; HAdV-B14 strains GZ02, not strain GZ01, is the first HAdV-B14 isolate emergent in China. Finally, we successfully applied this method for the whole-genome sequencing of two additional clinical isolates, HAdV-C2 (strain HK91) and HAdV-E4 (strain HK35), and the complete genomic sequences were acquired and annotated.

## Materials and Methods

### Cells and Virus Isolates

HAdV-B14 strain GZ01 was isolated from a throat swab of a 17-month-old child hospitalized with acute suppurative tonsillitis in Guangzhou, China (October 2010) (Zhang et al., 2017). HAdV-B14 GZ02 was isolated from a throat swab of a child hospitalized with bronchopneumonia in another hospital in Guangzhou (September 2010). Strains HK35 and HK91 were isolated from throat swabs of children with ARD in Hong Kong (2014). The throat swabs were inoculated into A549 cells (ATCC), respectively, and grown in Dulbecco’s minimum essential medium supplemented with 100 IU/ml penicillin, 100 μg/ml streptomycin, and 2% (v/v) fetal calf serum, at an atmosphere of 5% (v/v) carbon dioxide. The cells were observed for 10 days for cytopathic effect (CPE) and harvested for viral genomic DNA extraction. This study was approved by the Institutional Review Board of Southern Medical University following the Declaration of Helsinki, with the patient consent for using leftover specimens waived.

### Extraction of Adenoviral Genomic DNA as the Direct WGS Template

Viral genomic DNA was extracted from infected cells for genome sequencing using the Hirt viral genomic DNA extraction method modified by us (Hirt, 1967). In brief, a sample of each stock virus was grown in the A549 cells in a 75-cm^2^ tissue culture dish (Corning) at 37°C and an atmosphere of 5% CO_2_. The cells were infected following standard procedures at an approximate multiplicity of infection (MOI) of 10 and observed for CPEs every day. First, when an 80% CPE or more occurred, the supernatant was discarded, and 800 μl of the lysate [0.6% sodium dodecyl sulfate (SDS), 0.01 M ethylenediaminetetraacetic acid (EDTA), 0.01 M Tris–HCl (pH 7.4)] and 20 μl protease K were added to the cells. The mixture was incubated at 55°C for 1 h. If the cells detached before adding the lysate, the cells and medium were transferred to another centrifuge tube, centrifuged for 2 min at 1,000 × *g*, 4°C. Then, the supernatant was discarded, and 2.4 ml of lysate and 60 μl protease K (TAKARA, 20 mg/ml stock solution) was added to the centrifuge tube, then gently mixed by a pipet. The mixture was transferred to the original dish and incubated at 55°C for 1 h. Next, the cell lysis was transferred to 1.5-ml centrifuge tubes (800 μl per tube). Three hundred microliters of 5 M NaCl was added to each tube (the volume of NaCl vs. lysis: 3:8) and mixed gently by inversion (≥10 times). The tubes were placed at 4°C overnight (≥12 h) and then centrifuged at 1,7000 *g*, 4°C for 20 min. The supernatant was collected into another fresh 1.5-ml centrifuge tube, and viral DNA was isolated from the supernatant solution by standard phenol/chloroform/isoamyl alcohol extraction and ethanol precipitation. The precipitated DNA was resuspended in 50 μl of TE buffer, and the genomic DNA was quantified by electrophoresis gel.

Prior to sequencing, we performed restriction endonuclease analysis (REA) to check and identify the genomic DNA rapidly. *Eco*RI was used, and an *in silico* REA map was generated first by the Vector NTI 11.5.1 software (Invitrogen Corp., San Diego, CA, United States) (Zhao et al., 2014; Yu et al., 2016; Zhang et al., 2017; Pan H. et al., 2018; Yan et al., 2020a). Then, the genomic DNA was digested by *Eco*RI according to the instruction.

### HAdV Genome Sequencing Strategy

The isolates’ genome sequences were obtained and used as the sequencing template by the Sanger primer-walking sequencing method without PCR amplification. To verify the method’s accuracy, the HAdV-B14 strain GZ01 (JQ824245) stored in our laboratory was sequenced again, previously sequenced by traditional first-generation sequencing (Zhang et al., 2017). Using the strain GZ01 sequence (JQ824245) as a template, all one-way sequencing primers for HAdV-B14 sequencing were designed. The distance between the two sequencing primers was 500–600 bp. For other unknown isolates, to determine the genomic reference sequence for primer design, molecular typing of HadVs has been performed first using the universal PCR primers revised according to our previous study (Han et al., 2013): HexonF (5′GCCCCARTGGGCRTACATGCACATC3′) and HexonR (5′AGCACSCCSCGRATGTCAAAG3′). The amplification length was 300 bp, and the amplification conditions were as follows: 94°C for 1 min, 34 cycles of 94°C for 30 s, 55°C for 30 s, and 72°C for 20 s with a final extension of 72°C for 7 min. The products were subsequently sequenced and analyzed. By blast, the reference strains (GenBank nos. JQ824845, KX384951, and JX173077) were selected as the primer-design templates for HAdV-B14, HAdV-E4, and HAdV-C2, respectively. The sequencing primers covering the whole genomes were designed and synthesized for all three types (Supplementary Table 1).

**FIGURE 1:**
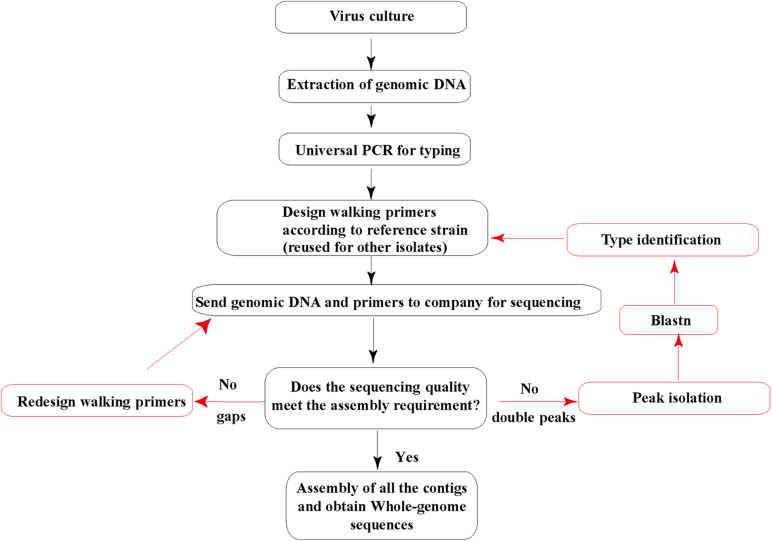
Human adenovirus (HAdV) genome sequencing strategy. The genomic DNA extracted and purified is sent to a sequencing company along with all the walking primers. Red arrows indicate additional sequencing cases that may occur.

Then, the genomic DNA and sequencing primers were submitted to Invitrogen (Guangzhou) for Sanger sequencing. All the sequencing was repeated three times. DNA sequence fragments were assembled using the SeqMan software from the Lasergene package (DNA Star; Madison, WI) into a single contig for each strain. The default parameters of SeqMan were used during the assembly: trim sequence ends, removed contaminant sequences, optimized sequence assembly order, medium quality stringency, and sequence percent match higher than 90% in contigs. Each sequence from Sanger sequencing assembled into contigs should be more than 600 nucleotide acid long with clear single peaks. Most of the base QV should be higher than 40, and the signal/noise is higher than 30. Suppose the sequences from Sanger sequencing contain double peaks with similar and high signals. In that case, we will do the peak isolation and blast the two separated sequences and confirm if they are mixed adenovirus isolates, i.e., coinfection with two types of HadVs. If there are gaps among contigs after assembly, a couple of walking primers will be redesigned and sent for additional sequencing (Figure 1).

### Direct Sequencing of the Inverted Terminal Repeat Ends

The sequencing primers for the 5′ and 3′ ends of the linear human adenovirus genome [inverted terminal repeat (ITR)] are designed according to the obtained contig. Forward and reverse sequencing primers were 200–300 bp away from both genome ends, respectively. Both 5′ and 3′ ends, including inverted terminal repeats, were sequenced directly using genomic DNA as a template by Sanger primer-walking sequencing method described earlier (Zeng et al., 2016; Cheng et al., 2018).

### Genome Annotation and Sequence Analysis

The fragment sequences were collected and assembled with SeqMan software from the Lasergene package (DNAStar). Genome annotation provided an additional layer of sequence quality control. Unresolved and ambiguous sequences were resequenced with primers close to the regions in question.

### Phylogenetic Analysis

Phylogenetic analysis of the whole genomes was performed using MEGA X^2^, and maximum composite likelihood method was applied to generate neighbor-joining and bootstrapped phylogenetic trees with 1000 bootstrap replications; all other parameters were set by default (Jing et al., 2019; Zhang et al., 2019; Yan et al., 2020b).

## Results

### Extraction of Adenoviral Genomic DNA

**FIGURE 2:**
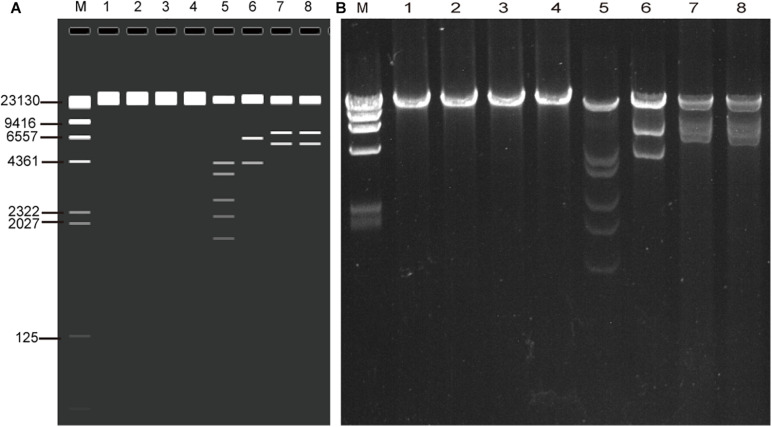
Agarose gel electrophoresis of the genomic DNA of four adenovirus isolates (HK91, HK35, GZ01, and GZ02) and restriction enzyme analysis of the genomes **(A)** *in silico* and **(B)** wet bench. MW, *λ* DNA/*Hin*dIII marker; lane 1, HK91 genome; 2, HK35 genome; 3, GZ01 genome; 4, GZ02 genome; 5–8, four genomes + *Eco*RI digestion. The predicted molecular weights of digested fragments of HK91 are 21,360, 4,294, 3,673, 2,670, 2,218, and 1,739 bp; of HK35 are 25,289, 6,404, and 4,273 bp; and of GZ01 and GZ02 are 21,917, 7,032, and 5,815 bp.

The whole genomic DNA of four isolates (HK35, HK91, GZ01, and GZ02) were extracted by the improved genomic DNA extraction method successfully. Viral genomic DNA (40–75 μg) was extracted from a 10-cm cell culture dish, and the OD_260_/OD_280_ and OD_260_/OD_230_ ratios were around 1.9 and 2.2, respectively. This indicated that the DNA extracted by this method was not contaminated by proteins, carbohydrates (sugars), salts, or organic solvents. The quality and quantity is high enough to meet our sequencing requirements. To verify if the viral genomic DNA obtained is correct or not, the genomes were digested with *Eco*RI restriction endonuclease. The restriction maps generated were consistent with the *in silico* restriction maps predicted by the Vector NTI 11.5.1 software (Figure 2).

### Sequencing and Analysis of Both Inverted Terminal Repeats

**FIGURE 3:**
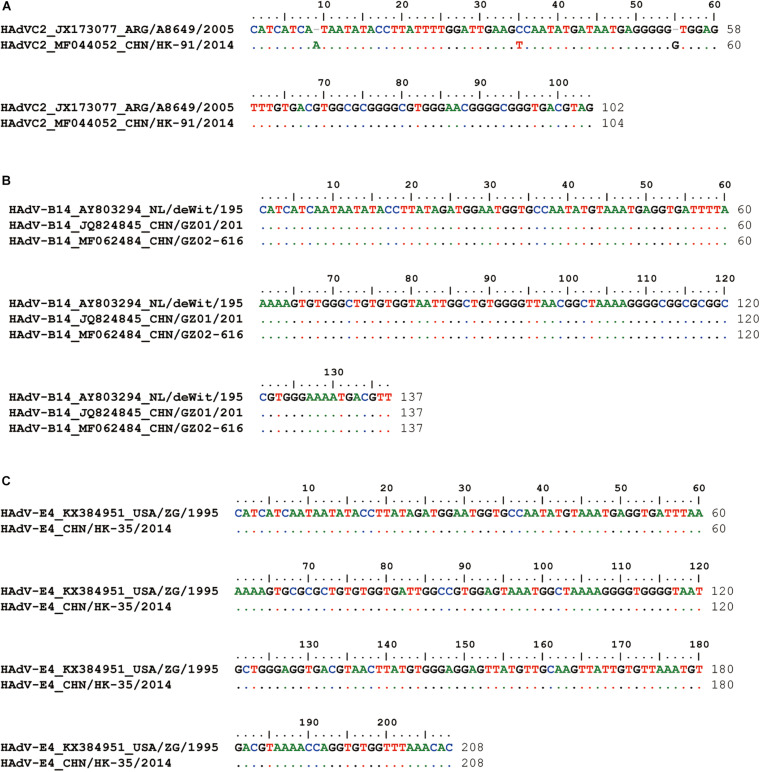
Inverted terminal repeat (ITR) alignment between reference strains and clinical isolates **(A)** HK91, **(B)** GZ01 and GZ02, and **(C)** HK35. HAdV-C2 reference strain: A8649 (JX173077); HAdV-B14 reference strain, deWit (AY803294); HAdV-E4 reference strain, ZG (KX384951). Different colors represent different base types.

Compared with the standard PCR and Sanger sequencing method, it is hard to get the complete ITR sequences; our novel sequencing method can easily acquire the accurate adenoviral end sequences (Figure 3). HK91 ITR is 104 bp, both GZ01 and GZ02 ITRs are 137 bp, and HK35 ITR is 208 bp. All the ITR sequences are about the same length as the reference strains after the alignment (Figures 3A–C). However, we still found two nt deletions in the ITR sequence of HAdV-C2 reference strain ARG/A8649/2005. The sequencing error of the ARG/A8649/2005 strain was speculated, and it could be further confirmed using genomic DNA as a direct template for Sanger sequencing.

### Comparative Genomic Analysis of the Four HAdV Isolates

**FIGURE 4:**
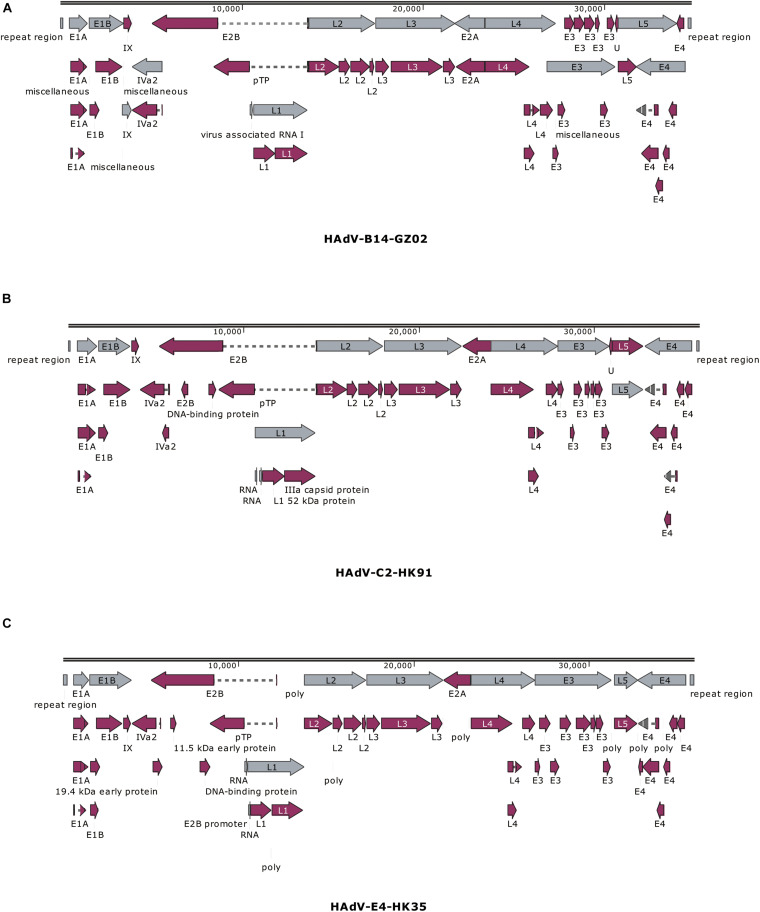
Transcriptional maps and genome organizations of HAdV-B14 isolates **(A)** GZ01/GZ02, **(B)** HAdV-C2 isolate HK91, and **(C)** HAdV-E4 isolate HK35. The genome is indicated by two black horizontal lines marked at 10,000-bp intervals. The gray arrows indicate the early, intermediate, and late transcription units; the magenta arrows indicate coding regions. Arrows reflect the direction of the coding transcripts.

The previous study has found that some adenovirus isolates had partial gene deletion (Marinheiro et al., 2011; Su et al., 2011; Pan Q. et al., 2018; Shen et al., 2020). For example, both Marinheiro et al. (2011) and Su et al. (2011) reported HAdV-7 variants in which E3 regions were partially deleted. Therefore, we analyzed the transcriptional maps and genome organizations of HAdV-C2 (HK91), HAdV-B14 isolates (GZ01/GZ02), and HAdV-E4 (HK35) (Figure 4). They could be used to visually identify if there were gene deletions or insertions in the genomes.

The map of GZ02 was the same as GZ01. A total of 38 coding sequences were identified (Figure 4A). These genome data of strain GZ02 were deposited in GenBank (accession number MF062484) under the formal name preferred by the National Center for Biotechnology Information (NCBI) (Seto et al., 2011): “Human adenovirus 14 isolate HAdV-B/CHN/GZ02/2010/14[P14H14F14].” The genome comprises 34,767 bp, with a GC content of 48.83%, consistent with the other subspecies B2 (mean of 49%). These lengths are very similar to that of other HAdV-B14p1 strains 303600 (Lackland Air Force Base, United States; 2007) and CHN2012 (Beijing, China; 2012), as well as the prototype from 1955: genome sizes of 34,763, 34,760, and 34,764 bp, respectively. HAV-B14 strain GZ02 was isolated in September 2010, 1 month earlier than strain GZ01. Surprisingly, both isolates had identical genomic sequences, indicating that both isolates have the same origin. Given that both strains were isolated from two children from different hospitals in Guangzhou, the transmission chain of HAdV-B14 needs to be further explored.

The genomic sequence of HAdV-C2 strains HK91 was annotated and deposited into GenBank under accession number MF044052. Like other genera Mastadenovirus, the genome of strain HK91 was organized into early, intermediate, and late transcription regions (Figure 4B). The genome was 35,954 bp in length and had an overall base composition of 23.17% A, 27.95% C, 27.24% G, 21.61% T, and 55.2% GC. Thirty-nine protein-coding sequences and two RNA-coding sequences were identified in the genomic sequence.

The genomic sequence of HAdV-E4 strains HK35 was annotated and deposited into GenBank under accession number MW692349. The genome was 35,966 bp in length and had an overall base composition of 22.65% A, 28.35% C, 27.93% G, 21.07% T, and 56.28% GC. The genome was divided into early, intermediate, and late transcription regions (Figure 4C). A total of 75 features are annotated, including gene, CDS, RNA, poly(A), or poly(T).

### Phylogenetic Analysis of Fiber, Hexon, Penton Base Genes, and the Whole Genomes

**FIGURE 5:**
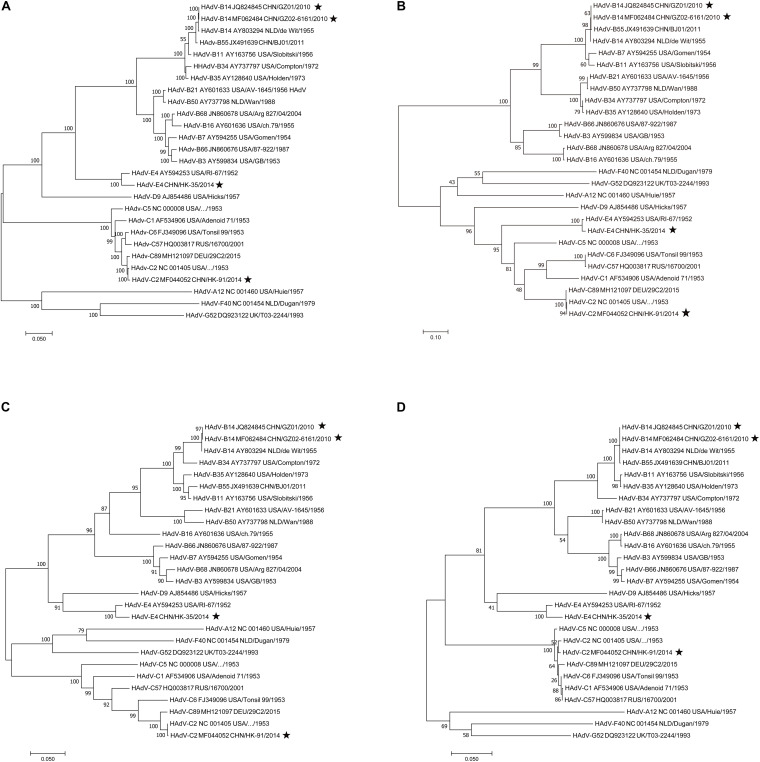
Phylogenetic analysis of the **(A)** whole genomes, **(B)** fiber, **(C)** hexon, and **(D)** penton base genes. Nucleotide sequences are from prototype strains in different types available from GenBank except for the four isolates (★). Taxon names include GenBank accession number, isolation country, strain name, and year of isolation. Phylogenetic trees were generated using the neighbor-joining method with 1,000 replicates and constructed by the MEGA X software. In these analyses, default parameters were applied, with a maximum-composite-likelihood model. Bootstrap numbers shown at the nodes indicate the percentages of 1,000 replications producing the clade, with values above 80 considered robust. The scale bar is in units of nucleotide substitutions per site.

To find potential recombination that is usually emergent among the hexon, fiber, and penton base genes, the phylogenetic analysis of the fiber, hexon, penton base genes, and the whole genomic sequences was performed using MEGA X. Phylogenetic analysis of the HAdV-B14 whole genomes (Figure 5A) shows that the recent HAdV-B14 isolates (strains GZ01 and GZ02) are closely related to each other as well to the earlier reported HAdV-B14p genome (strain de Wit, 1955). By comparing the phylogenetic trees of fiber (Figure 5B), hexon (Figure 5C), and penton base genes (Figure 5D) of strains GZ01/GZ02, HK91, and HK35, no recombination was identified in these isolates. All the four isolates formed subclades with their prototype strains. However, new recombinant variants could be identified by applying this method to screen the clinical samples during the ARD surveillance (see section “Discussion”).

## Discussion

In this study, three HAdV isolates (HK35, HK91, and GZ02) were isolated and cultured from throat swabs of clinical children with respiratory diseases in Hong Kong and Guangzhou. CPE were all found in A549 cells 5 days postinoculation. The complete genomes were extracted and sequenced by Sanger primer-walking method without any PCR amplification, which ensures the sequencing accuracy. The complete sequences of the ITRs were also obtained without PCR. The whole genomic sequence of HAdV-B14 strain GZ01 was resequenced using this method. It was identical to that sequenced previously with PCR plus Sanger method (GenBank no. JQ824845), which suggested that our HAdV sequencing method is highly accurate.

A HAdV-B14 variant, 14p1, emerged in the United States since 2005 and caused multiple outbreaks in both civilian and military settings and led to 76% hospitalization rate and 18% fatality rate (Binn et al., 2007; Centers for Disease Control and Prevention [CDC], 2007; Metzgar et al., 2007; Louie et al., 2008; Lewis et al., 2009). Since 2009, this genotype has caused at least two ARD outbreaks in Europe and one outbreak in Canada (Carr et al., 2011; O’Flanagan et al., 2011; Parcell et al., 2014). There were no HAdV-B14 cases in China until October 2010, when HAdV-B14 (strain GZ01) emerged in Guangzhou (Zhang et al., 2012b, 2017). Subsequently, there were at least three additional HAdV-B14-related ARD outbreaks in China: 43 students with ARD in an elementary school in Gansu Province (2011) (Huang et al., 2013), 30 adults presented with severe symptoms that required hospitalization in Beijing (2012) (Mi et al., 2013; Tang et al., 2013), and 24 students in a middle school in Liaoning Province (2012) (Yu et al., 2014). In our study, strain GZ02 isolated in September 2010 had an identical genomic sequence with strain GZ01, which was identified as the first HAdV-B14 strain emergent in China (Zhang et al., 2012b, 2017). However, strain GZ02 was isolated 1 month earlier than strain GZ01. Therefore, we have identified that the first HAdV-B14 isolate emergent in China is strain GZ02 (September 2010), not strain GZ01.

HAdV-C2 was previously reported as an uncommon type that causes asymptomatic or mild and self-limiting disease. However, it could lead to a severe outcome in immunocompromised hosts (Lion, 2014). Recently, more and more type 2 adenoviruses were identified in pediatric infectious disease (Ma et al., 2015; Dhingra et al., 2019), some of which were recombinants with other members in HAdV-C (Wang et al., 2016; Dhingra et al., 2019; Zhang and Huang, 2019; Zhao et al., 2019). In this study, the complete genomic sequence of HAdV-C2 strain HK91 was obtained by our method. The genome was highly similar to that of another strain isolated in Xizang, China (99.56%).

Given that more and more respiratory cases caused by HAdV-C are emergent, further surveillance in HAdV-C pathogens is highly necessary and recommended. Although no recombination was found in these genomes, the sequencing and analysis procedure will reference clinicians and clinical laboratories and be used for other isolates in the future. Our continuous surveillance found a recombinant HAdV strain by sequencing the whole-genome sequence using this method and the phylogenetic analysis of the penton base, hexon, and fiber genes (data not shown). The penton base gene is closer to HAdV-1, but both the hexon and fiber genes are closer to HAdV-2. This strain’s genomic sequence has been submitted to NCBI and HAWG to identify as a new genotype.

HAdV-E4 is a zoonotic pathogen that contains the chimpanzee adenovirus genome. It was typically restricted to United States military populations with occasional infections in civilian populations (Kajon et al., 2018). The “old” HAdV-E4 lack a critical replication motif in ITRs, nuclear factor I (NF-I), found in all HAdV respiratory pathogens, which is a host transcription factor that binds to 23–36 nt of the HAdV origin of replication (Mul et al., 1990; Hatfield and Hearing, 1991) and is recruited by the human adenoviral replication complex (Nagata et al., 1983). However, in our earlier study, we found that the re-emergent HAdV-E4 pathogens (strain HK35 and other recent HAdV-E4 isolates) circulating in children obtained NF-I in ITRs by recombination with other human adenoviruses (Zhang et al., 2019), which may facilitate HAdV-E4 to adapt hosts more efficiently. Attention should be paid to this recombinant HAdV-E4 circulating in civilian populations.

Our sequencing method improves the sequencing accuracy greatly because the genomic DNA is used as a direct sequencing template, and no PCR amplification is involved, which will avoid the base mismatch. Additionally, many ITR sequences released in GenBank are inconsistent at the left and right ends due to sequencing errors. Our method could be used to obtain the complete and accurate ITR sequences at the 5′ and 3′ ends of the genomes (Zhang et al., 2012a,b, 2017, 2019; Zhao et al., 2014; Zeng et al., 2016; Jing et al., 2019). The sequencing primers can be reused for the same type. Compared with the NGS method, our sequencing method could be applied for one or several clinical strains. More importantly, the gap that could not be sequenced by the second-generation sequencing but can be sequenced by our method; no PCR amplification needed.

Using the improved genomic DNA extraction protocol in our study, we obtained the HAdV genomic DNA with high quantity and quality to be used as a direct sequencing template. Li et al. (2016) compared the three methods for the adenoviral genomic DNA extraction. They found that when compared to the traditional Hirt’s method, Viral DNA/RNA Extraction Kits A and B could be used to extract high-quality adenovirus genomic DNA. However, usually if kits are used, the cellular genomic DNA is not easy to be eliminated, which may interfere our WGS in this study. Further verification would be necessary. In our study, the sequencing data were specific, and no background noise from hosts was found. The genomic DNA is also suitable for genotyping HAdVs by restriction enzyme analysis (Zhang et al., 2016). When the sequencing primers could not work due to the genome variation and poor primer binding, new primers are needed for primer-walking sequencing, which may be a little time consuming but guarantee genomic accuracy.

In conclusion, in this study, the sequencing method for the complete or partial genomes is universal, accurate, and convenient. It is an option for the genome sequencing of common human adenoviruses, especially when the samples include recombinant adenoviruses and accurate sequences are in need. The sequencing strategy may also be applied to the WGS of the other DNA viruses.

## Data Availability Statement

The datasets presented in this study can be found in online repositories. The names of the repository/repositories and accession number(s) can be found below: GenBank, MF062484, MF044052, and MW692349.

## Author Contributions

QZ conceived and designed the experiments. JZ and ZY collected the clinical samples. SZ, WG, KM, YY, JO, and JZ performed the experiments. SZ and QZ analyzed the data. SZ, JW, and QZ contributed to the preparation of the manuscript. All authors contributed to the article and approved the submitted version.

## Conflict of Interest

The authors declare that the research was conducted in the absence of any commercial or financial relationships that could be construed as a potential conflict of interest.

## Footnotes

1 http://hadvwg.gmu.edu

2 http://www.megasoftware.net
